# Supplementary material for: Fear the reaper: ungulate carcasses may generate an ephemeral landscape of fear for rodents
Source: R Soc Open Sci. 2020 Jun 3;7(6):191644. doi: 10.1098/rsos.191644 (PMC7353961; doi:10.1098/rsos.191644)
Supplement: Figures S1-S4, Tables S1-S2 [file rsos191644supp1.docx]

**Electronic Supplementary Material**

**Figure S1.** An overview of changes in percent cover of vegetation, carcass, and stone from 2017 to 2018. As expected, stone and carcass depict the smallest changes, and this may be due to slight shifting of quadrats or realized changes in vegetation cover around stones and/or carcass decomposition (spreading of material), respectively. Plant, moss, lichen, grass, and soil depict the largest changes in percent cover. No cover type changed more than 60% (max change was soil cover). The background is a kernel density of the carcasses, with darker areas representing higher carcass density.


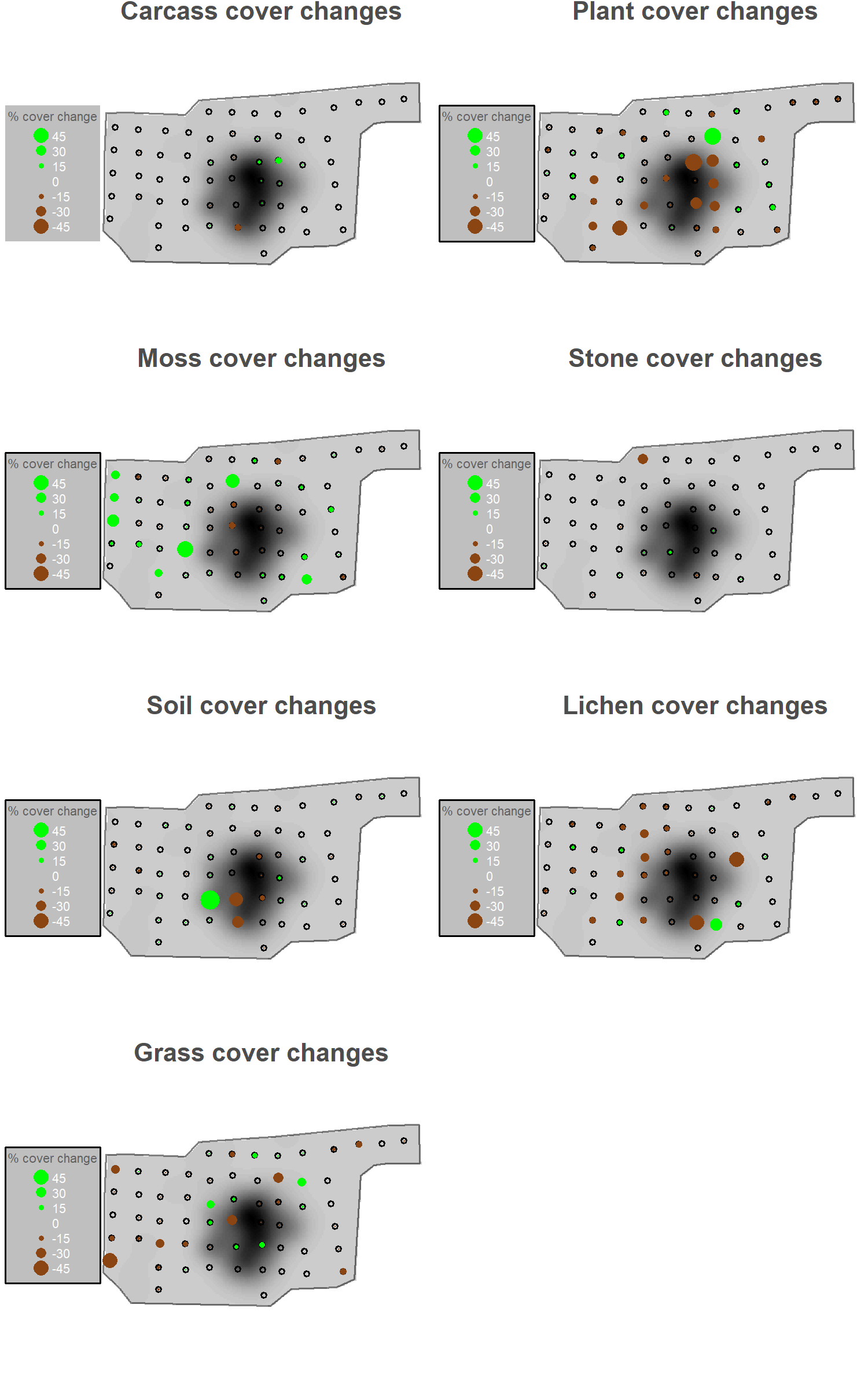


**Figure S2.** Semivariograms of response variables (rodent presence, bird abundance, mesopredator abundance, and changes in rodent presence-absence) in relation to the spatial configuration of sample plots (‘dist’: distance lags in meters). Solid lines are loess curves plotted scatter plots. We constructed a neighbour network based on the minimum possible distance (12 meters; vertical dashed line), i.e. at which no ‘empty neighbour sets’ occurred. We used this network test and account for spatial autocorrelation of model residuals and incorporated this spatial structure as a residual autocovariate into model structures prior to model selection when appropriate, i.e. when a Moran’s I test of model residuals depicted a significant pattern (*P*-value < 0.05).


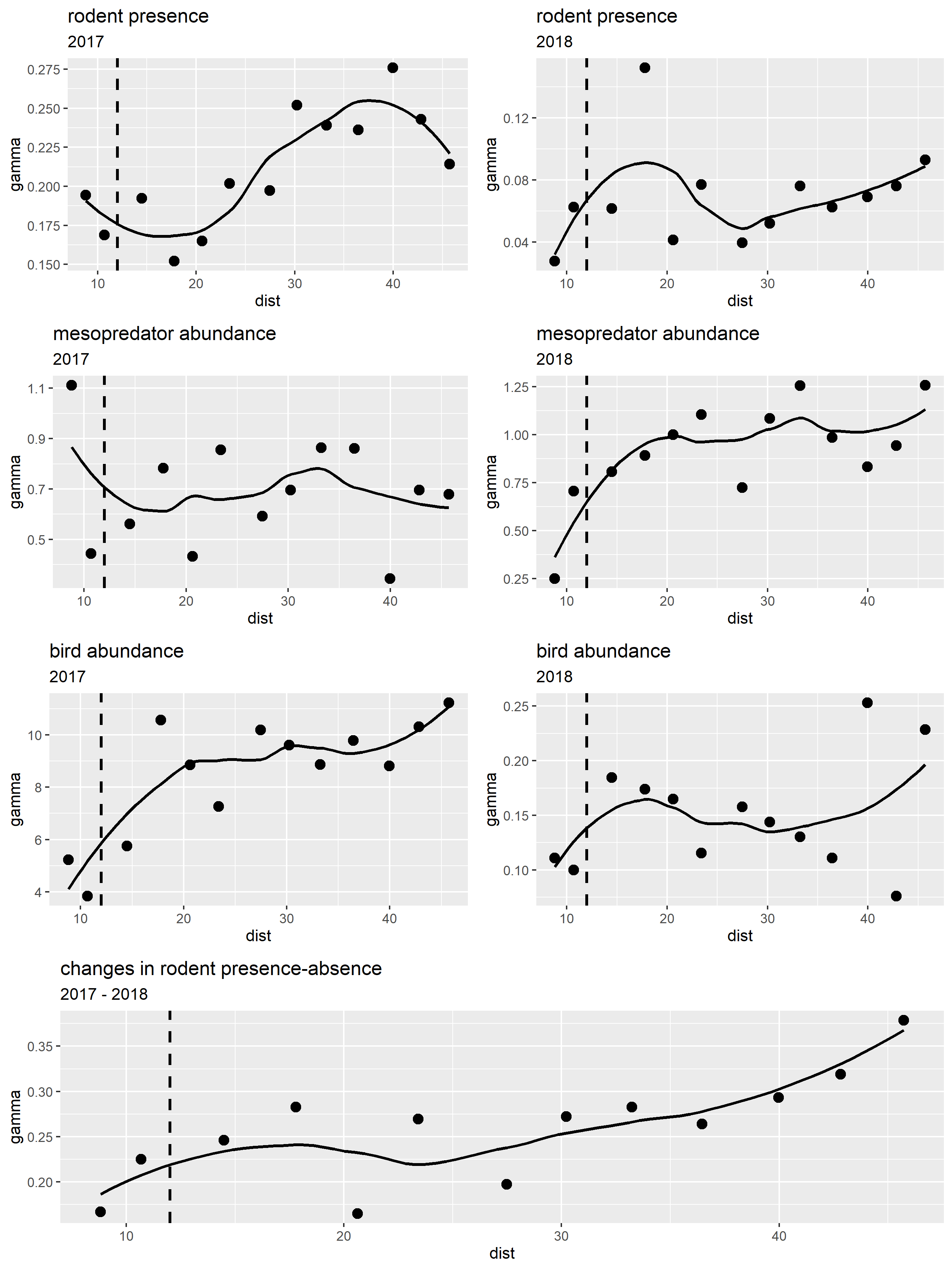


**Figure S3.** Predictions from the most parsimonious model for explaining observed changes in rodent presence-absence (from absence to presence; from presence to absence; and no change: remains present or absent across years) in 1 × 1 meter plots. The most parsimonious model only included the covariate for interannual changes in bird use of the study area, with the next closest model of a ΔAICc > 2 (Table S3). As bird use decreases, the probability of rodents becoming present increased significantly (*p*-value < 0.0429; right panel). Conversely, the probability of no change in rodent presence-absence was highest when bird abundance did not change or slightly increased, but this increase in bird abundance was minimal (middle panel). As a change from presence to absence occurred only once in our dataset, there is too much uncertainty around its prediction in relation to changes in bird abundance, but this occurred where birds were once quite abundant and then disappeared (left panel). Areas shaded in green are the predictions and confidence intervals over observed ranges of the data for each response level, whereas areas shaded in grey are predictions and confidence intervals across the minimum and maximum observed values over the whole study site.Note: the majority of changes in rodent presence-absence depicted either no change or a positive change with only one observation for a negative change.


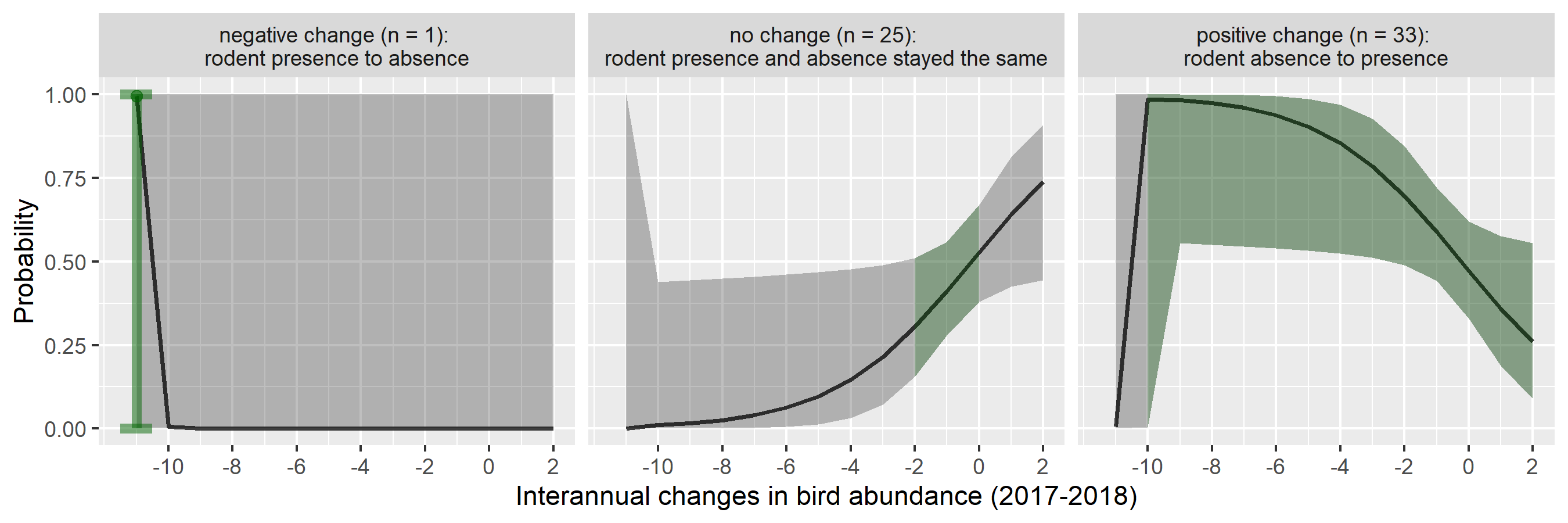


**Figure S4.** A typical example of an image captured from the carcass study site during summer-fall seasons in 2017 and 2018. In 2017, raven were numerous (marked by yellow arrows; N = 22), whereas they were sparse and infrequent in 2018. Although not quantified here, this image helps put into context the level of predation risk that rodents likely incurred during 2017 as compared to 2018.


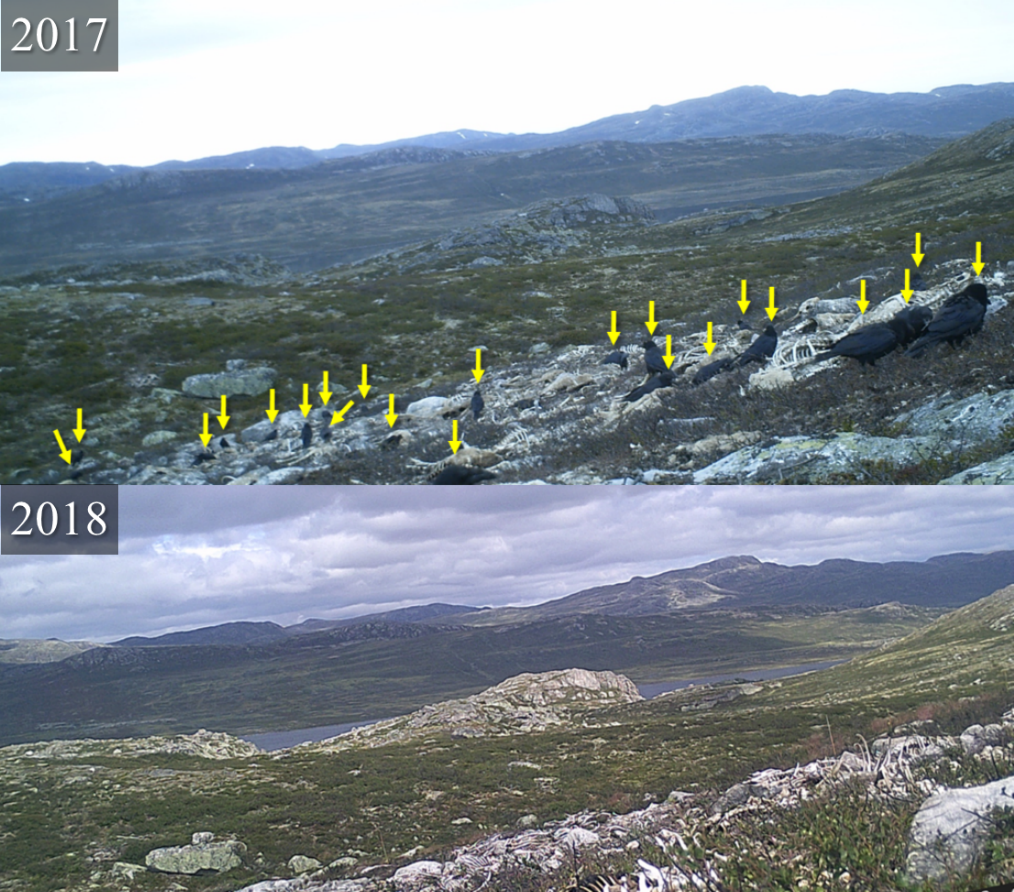


**Table S1.** Model selection results using Akaike’s Information Criterion with a second-order bias correction (*AICc*) for generalized linear models fitted to rodent presence, the abundance of bird and mesopredator feces, and for multinomial logistic regression models fitted to data depicting changes to rodent presence in relation to changes in mesopredator and bird abundance, and percent changes in cover types. Only models with a *ΔAICc* ≤ 4 are shown along with model structure, degrees of freedom (*df*) and model weights (*w*). Search radii used for calculating “carcass density” variables are shown in square brackets and the year 2017 was set as the reference. “Bird” and “mesopredator” are changes in the abundance of avian and mammalian mesopredator faeces, respectively, from 2017-2018 and were counted within 1 × 1 m^2^ plots. “Carcass-”, “soil-“, “lichen-”, and “plant cover” refer to changes in the percent coverage in 1 × 1 m^2^ plots by carcass (skin, tissue, hair, bone, and/or flesh), bare soil, lichen, and herbaceous plants, respectively. The residual autocovariate term (RAC) was only applied to bird and mesopredator responses, as they were the only model residuals depicting spatial autocorrelation from a Moran’s I test. All model variables are continuous except for ‘year’ which was treated as a categorical factor. The most parsimonious models are highlighted grey and in bold. Note that ‘bird’ is included in every model structure predicting changes to rodent presence-absence.

**Table S2.** Model diagnostics using goodness-of-fit (GOF) tests, McFadden’s Pseudo-R^2^, and Moran’s I test, on model residuals to detect whether spatial autocorrelation of the sampling design significantly affected results (P-value < 0.05). There was no indication of spatial autocorrelation in any of the model residuals following the inclusion of the residual autocovariates in bird and mesopredator abundance models. *Note: the lack of fit for changes in rodent presence-absence results in the under-prediction of ‘positive change’ and an over-prediction of ‘no change’, which is due to the response level ‘negative change’ having only one observation. If that observation and the response level is dropped, interpretation remains the same for the directional effect from changes in bird abundance on the probability of a positive change in rodent presence-absence, but effect sizes and standard errors are different, and the GOF test becomes non-significant.
